# Big data, big consortia, and pain: UK Biobank, PAINSTORM, and DOLORisk

**DOI:** 10.1097/PR9.0000000000001086

**Published:** 2023-08-10

**Authors:** Harry L. Hébert, Mathilde M.V. Pascal, Blair H. Smith, David Wynick, David L.H. Bennett

**Affiliations:** a Chronic Pain Research Group, Division of Population Health and Genomics, Ninewells Hospital & Medical School, University of Dundee, Dundee, United Kingdom; b Neural Injury Group, Nuffield Department of Clinical Neuroscience, John Radcliffe Hospital, University of Oxford, Oxford, United Kingdom; c Bristol Medical School, University of Bristol, Bristol, United Kingdom

**Keywords:** UK Biobank, DOLORisk, PAINSTORM, Chronic pain, Neuropathic pain, Genetics, Epidemiology

## Abstract

Chronic pain (CP) is a common and often debilitating disorder that has major social and economic impacts. A subset of patients develop CP that significantly interferes with their activities of daily living and requires a high level of healthcare support. The challenge for treating physicians is in preventing the onset of refractory CP or effectively managing existing pain. To be able to do this, it is necessary to understand the risk factors, both genetic and environmental, for the onset of CP and response to treatment, as well as the pathogenesis of the disorder, which is highly heterogenous. However, studies of CP, particularly pain with neuropathic characteristics, have been hindered by a lack of consensus on phenotyping and data collection, making comparisons difficult. Furthermore, existing cohorts have suffered from small sample sizes meaning that analyses, especially genome-wide association studies, are insufficiently powered. The key to overcoming these issues is through the creation of large consortia such as DOLORisk and PAINSTORM and biorepositories, such as UK Biobank, where a common approach can be taken to CP phenotyping, which allows harmonisation across different cohorts and in turn increased study power. This review describes the approach that was used for studying neuropathic pain in DOLORisk and how this has informed current projects such as PAINSTORM, the rephenotyping of UK Biobank, and other endeavours. Moreover, an overview is provided of the outputs from these studies and the lessons learnt for future projects.

## Abstract 2

Current challenges in understanding chronic pain pathogenesis are being overcome by the creation of large consortia and biorepositories with a harmonised approach to phenotyping.

## 1. Introduction

Chronic pain (CP), defined as pain lasting more than 3 months, represents a major global burden for years lived with disability and the associated economic impact due to health resources used and work absenteeism.^128^ As an example, nonspecific low back pain (LBP) is the largest single cause of years lived with disability globally,^49^ accounting for 11% of the entire disability burden from all diseases. In 2017, LBP was estimated to cost the UK up to 116 million lost workdays and approximately £12.3 billion through direct health care costs, production losses, and informal care https://www.gov.uk/government/publications/chronic-pain-in-adults-2017.

Pain has long been considered solely as a symptom, and it is only recently that CP has been recognised by the World Health Organization International Classification of Diseases (ICD)-11^121^ as a long-term condition in its own right.

Chronic pain prevalence increases with age as predisposing conditions such as obesity, arthritis, diabetes mellitus, and malignancy become more common.^41^ A recent meta-analysis of population-based epidemiological studies worldwide reported a pooled CP prevalence estimate of 31%,^111^ with an equivalent figure from UK studies of 43.5%,^41^ similar to data arising from UK Biobank (UKB, 42.9%).^75^ Of those reporting CP, a subset conservatively estimated at 20%^20^ have disabling CP that substantially interferes with activities of daily living. Similarly, there is an overlapping population who express a substantial need for health care. Such individuals are often characterised by comorbid depression, fear, cognitive dysfunction, avoidance of movement, and poor coping skills.^15^ From a public health perspective, the challenge is to prevent the progression of mild or transient pain to CP which becomes severe.^14^

The development and severity of CP involve a complex interaction between genetic, environmental, and clinical factors in vulnerable individuals.^126^ Chronic pain is heritable: Twin^72,78,110^ and extended family^61^ studies have provided estimates of 30% to 76%. Mutations in specific genes (most of which encode ion channels) cause rare (Mendelian) pain conditions in humans.^12^ This includes the disorder inherited erythromelalgia (characterized by pain and erythema of the extremities exacerbated by warming), which is caused by autosomal dominant and highly penetrant gain of function mutations in the gene *SCN9A* encoding the voltage-gated sodium channel Na_V_1.7.^13^ Human pain complex trait genetics (especially when combined with rich phenotypic data, biosamples, and large-scale brain imaging cohorts^34^) has the potential to revolutionise our understanding of CP pathogenesis, risk factors, and the determinants of treatment responses. Current significant challenges and limitations to this approach relate to (1) a lack of precision in CP phenotyping with respect to the duration, location, intensity, and quality of pain as well as the temporal relationship to predisposing factors and comorbidities such as anxiety and depression and (2) the size of the existing CP cohorts which are limited and, therefore, studies are relatively underpowered. Key to overcoming these challenges is large consortia with a harmonised approach to pain phenotyping and nationwide biorepositories with a wide range of genetic and nongenetic data. There is now an increasing international effort to harmonise data collection which is likely to further inform clinical practice. One example is “INTEGRATE-Pain”. This is a joint initiative between the US National Institute of Health (NIH) and Innovative Medicines Initiative-PainCare which is developing consensus on overarching core outcome domain sets for clinical pain trials and clinical pain research (https://www.comet-initiative.org/Studies/Details/2083). Newer cohorts now available to study CP include DOLORisk,^93^ and this has informed the approach taken by current studies such as the rephenotyped UKB Chronic Pain (UKB CP) Cohort and PAINSTORM.

This review introduces these cohorts, provides an overview of their main outputs, and outlines the key lessons learned.

## 2. UK Biobank (chronic pain cohort)

The scope of UKB, which comprises 500,000 volunteers enrolled between 2006 and 2010 at ages 40 to 69 from across the UK, provides a unique opportunity to examine the epidemiology and genetics of CP in a prospective population cohort. A brief assessment of CP was completed by all participants at booking (https://biobank.ndph.ox.ac.uk/showcase/field.cgi?id=6159) although it did not include validated questionnaires enabling categorisation of CP of diverse aetiologies. Using that initial data, we have previously demonstrated that the prevalence of CP and most regional (ie, site specific) musculoskeletal pains in UKB are similar to that found in other pain epidemiological studies. Our findings also reproduce known relationships from a range of socioeconomic and psychological factors.^75,90^

To address the lack of specificity when categorising CP, a UK academic consortium of clinicians and pain researchers, many of whom have experience of working with UKB^75,86,130,133^ and with a range of synergistic expertise in epidemiology, genomics, psychology, neuroimaging, and pain management, developed a UKB CP phenotyping survey (2017–2018). The pain phenotyping survey (https://biobank.ctsu.ox.ac.uk/crystal/ukb/docs/pain_questionnaire.pdf) was designed by a group of experts (including the authors B.H.S., D.W., and D.L.H.B.) and based on a series of validated questionnaires in routine use (Table 1), which were fully aligned with the CP cohorts described in this paper. These focused on the most prevalent causes of CP and associated comorbidities and risk factors.

**Table 1:** Questionnaires used in DOLORisk, PAINSTORM, and UK Biobank.

Category	Questionnaire	DOLORisk		PAINSTORM		UK Biobank	Reference
		Core	Extended	Core	Extended		
Demographics	Age, gender, years in education, working status, weight, and height	X	X	X	X	X	
	Ethnicity			X	X	X	
	Household income				X		
Characterisation of pain	Presence and duration of pain	X	X	X	X	X	
Family history	Family history of chronic pain		X		X		
Pain medication	Currently taking pain medication	X	X	X	X		
	Brief Pain Inventory—usefulness of medication		X		X		Cleeland and Ryan^30^
	Adherence to medication		X		X		
	Pain relief strategies other than medication				X		
Pain severity	Chronic pain grade	X	X	X	X		Von Korf et al.^127^
	Brief Pain Inventory—pain severity		X	Only the item on “pain on average”	X	X	Cleeland and Ryan^30^
Pain quality	DN4 Questionnaire	X	X	X	X	X	Bouhassira et al.^18^
	DN4 Examination		X		X		
	Neuropathic Pain Symptom Inventory		X		X		Bouhassira et al.^17^
	PainDETECT		X				Freynhagen et al.^47^
Pain location	List of locations	X	X	X	X		
	Body map		X		X		
Pain interference	PROMIS Pain Interference 8a		X				Amtmann et al.^3^
	PROMIS Ability to Participate in Social Roles and Activities				8a		Hahn et al.^54^
Pain-related worrying	Pain Catastrophizing Scale	X	X	X	X		Sullivan et al.^113^
Health status and psychological assessment	EQ-5D-5L	X	X	X	X	X	Herdman et al.^60^
	Patient Health Questionnaire-9					X	Kroenke et al.^67^
	PROMIS Depression	4a	8a	4a	8a		Pilkonis et al.^95^
	PROMIS Anxiety	4a	8a	4a	8a		
	PROMIS Sleep Disturbance	4a	8a	4a	8a		Buysse et al.^23^
	PROMIS Fatigue		8a		8a		Lai et al.^69^
	Fatigue Severity Scale					X	Krupp et al.^68^
	PROMIS Emotional Support	4a	4a	4a	4a		Hahn et al.^54^
	PROMIS Instrumental Support	4a	4a	4a	4a		
	Trauma	X	X	X	X		
	Ten-item Personality Inventory	X	X	X	X		Gosling et al.^51^
	International Personality Item Pool (Emotional Stability)		X				Goldberg^50^
	State Optimism Measure (SOM-7)				X		Millstein et al.^87^
Disease-specific (diabetic neuropathy)	Michigan Neuropathy Screening Instrument		X		X		Feldman et al.^42^
Lifestyle	Smoking	X	X	X	X		Campbell et al.^24^
	Alcohol	X	X	X	X		
	International Physical Activity Questionnaire		X		X		Craig et al.^33^
Bespoke questions	Financial situation and impact on pain management				X		
	Description of pain in the participant's own words				X		
Other chronic pain conditions	Fibromyalgia					X	Wolfe et al.^131^
	Headache and migraine					X	Lipton et al.^71^

DN4, Douleur Neuropathique en 4 Questions; EQ-5D-5L, EuroQol-5 dimensions-5 levels; PROMIS, Patient-Reported Outcomes Measurement Information System.

The CP phenotyping survey was then sent to ∼335,000 UKB participants who consented to recontact, had an email address, and were still actively participating as of May 2019 (ie, not deceased or withdrawn). The survey was (partially or fully) completed by ∼167,000 individuals (a response rate of 49.8%). Approximately 148,000 individuals either reported no CP (∼72,000), or CP (pain or discomfort that had been present for more than 3 months) and fully completed the Douleur Neuropathique en 4 (DN4) questionnaire (∼76,000; 51.1%). The data were released in early 2021 and are available to bona fide researchers worldwide. The UKB CP cohort has the added values of: (1) being by far the largest phenotyped CP cohort generated to date worldwide; (2) linkage to longitudinal GP records for >95% of respondents by 2024; (3) access to all the other rich datasets obtained by previous (and subsequent) UKB questionnaires completed by these individuals for imaging, depression, anxiety, cognition, multimorbidity, deprivation, etc; (4) the CP survey will be repeated in summer 2024 and extended to provide detailed outcome and treatment data on those with CP, thus allowing the identification over the intervening 5 years of those with newly reported CP; and (5) being of sufficient size and detail to allow data-derived categorisation of CP symptoms and risk factors.

The CP data demonstrate that 75% of subjects with CP reported pain having lasted for more than a year and about a third for more than 5 years. Using the Brief Pain Inventory (BPI) questionnaire, approximately 25% of subjects with CP reported severe or moderate pain whereas 20% reported severe or moderate interference in their activities of daily living. Over half of subjects with CP reported back or neck pain, over 40% had pain in one or more joints (the commonest being knee, then hip, hands, and feet), whereas 10% reported pain all over the body. The commonest self-reported CP diagnoses were osteoarthritis affecting one or more joints, followed by migraine, nerve damage or neuropathy, carpal tunnel, pelvic pain, and rheumatoid arthritis. Using the DN4 questionnaire, the prevalence of “possible” neuropathic pain (NeuP) was 9.2%, making up 18.1% of those with CP. Our recent analysis^7^ of those with NeuP demonstrated that this was significantly associated with worse health-related quality of life, having a manual or personal service type occupation and younger age compared with those without CP. As expected, NeuP was associated with diabetes and neuropathy but also with other pains (pelvic, postsurgical, and migraine) and musculoskeletal disorders (rheumatoid arthritis, osteoarthritis, and fibromyalgia). In addition, NeuP was associated with pain in the limbs and greater pain intensity and higher body mass index (BMI) compared with those with nonneuropathic pain.

### 2.1. Caveats/limitations

(1) Non-White ethnic backgrounds were rare in UKB (2.2%); 1.6% were from Black, Asian, and Minority ethnicities; 0.6% were mixed ethnicity; and the remaining 97.8% were White. This compares with 18.3% non-White in England and Wales in the 2021 Census.^35^ In the 2011 Census, which is closer to when the UKB cohort was recruited, 14% were non-White in England and Wales^36^ and 4% were non-White in Scotland^28^ (2022 census not yet available).
(2) There was an overrepresentation of participants who were female, of younger age, who had lower BMI, and who were less socially deprived in the group that completed the 2019 pain phenotyping questionnaire compared with the rest of the UKB cohort who did not. The overrepresentation of participants of younger age and who were less socially deprived could potentially be due to the fact that the questionnaire was only available online.
(3) The definition of NeuP relies on a self-completed screening tool, which does not meet the grading system for “probable” or “definite” NeuP. These necessitate clinical examination which is clearly not feasible in a large population survey.

### 2.2. Outputs

Because of the large sample size and range of data available compared with other cohorts, UKB allows associations to be quantified with greater precision and across different levels of demographics. As the data from the CP phenotyping survey was only released in early 2021, the results of studies using these data are only just beginning to emerge. Up to this point, studies have used the CP data that were collected at baseline recruitment. This has limited studies to specific single or multipain sites, without consideration for underlying aetiology. An extensive but nonexhaustive list of pain studies conducted either wholly or partially using UKB is provided in Table 2. These are intended to provide an overview of the kind of analyses that are possible using the cohort.

**Table 2:** Publications on (chronic) pain using the UK Biobank cohort.

Study	Pain phenotype	Design	Finding
Allen et al.^1^	AP and CP	CS	Social exclusion and loneliness are associated with pain
Atkins et al.^5^	CP	CS	Low cardiovascular disease score individuals had less chronic pain
Beasley et al.^8^	CWP	CS	Relationship between alcohol consumption and reporting of CWP
Beasley et al.^9^	CWP	MR	Protective effect of alcohol on CWP is not supported
Benavides et al.^10^	CP	Genetic association	rs1045642 (*ABCB1*) effect on response to chronic pain treatment with nortriptyline or morphine combo
Bortsov et al.^16^	BP (acute and chronic)	GWAS	13 GWS loci for chronic BP, none for acute BP. SNP heritability 4.6% for chronic BP and 0.8% for acute BP
Broberg et al.^21^	General pain and multisite CP	MR	Bidirectional causal relationship between insomnia and pain
Carvalho et al.^25^	Musculoskeletal pain	CS and longitudinal	T2D is associated cross-sectionally and longitudinally with shoulder or neck, knee, or hip pain and longitudinally with neck or shoulder pain
Carvalho et al.^26^	Musculoskeletal pain	CS	Metformin is protective of back, knee, neck or shoulder, and multisite pain
Cassidy et al.^27^	Drugs prescribed for CP	CS	Opiate and NP medications taken with cardiometabolic medications associated with obesity, increased waist circumference, and hypertension compared with cardiometabolic medications alone
Chen et al.^29^	Musculoskeletal pain	Longitudinal	Higher number of pain sites associated with risk of all-cause mortality
Cox et al.^32^	Opioid cessation/CBP	GWAS	PRS for opioid cessation significantly associated with chronic back pain, being a former drinker, and being a former smoker
Faber et al.^37^	Hip pain	CS	Cam morphology is associated with hip pain
Faber et al.^38^	Hip pain	CS	Radiographic hip osteoarthritis, total osteocyte area, joint space narrowing and acetabular, and superior and inferior femoral osteophyte areas were all associated with hip pain
Faber et al.^39^	Hip pain	CS	Osteophytes and joint space narrowing are associated with hip pain
Farrell et al.^40^	CP	GWAS/PhWAS	Shared genetic signature across 8 chronic pain types and 1492 biopsychosocial traits. 488 traits with causal association with CP
Freidin et al.^45^	BP	GWAS	3 loci associated with BP. Pleiotropic effects of genetic risk factors for BP, height, and intervertebral disk problems. Genetic correlations between BP and depression symptoms, neuroticism, sleep disturbance, overweight, and smoking
Freidin et al.^46^	Chronic BP	GWAS	2 and 7 GWS loci associated with chronic BP in males and females, respectively
Green et al.^53^	Frozen shoulder	GWAS/MR	5 GWS loci associated with frozen shoulder. Diabetes but not obesity is a causal risk factor for frozen shoulder
Hanlon et al.^55^	CWP	CS	Physical, sexual, and emotional childhood maltreatment and neglect associated with CWP
Hastie et al.^56^	CP and CWP	Longitudinal	CP and CWP associated with hospital admission for COVID-19. CWP but not CP associated with COVID-19 mortality
Jin et al.^62^	Oral inflammatory diseases (including mouth ulcer, painful gums, and toothache)	GWAS meta-analysis	31, 4, and 4 GWS loci associated with mouth ulcer, painful gums, and toothache. 2 novel GWS loci associated with painful gums and toothache
Johnston et al.^63^	Multisite CP	GWAS/MR	SNP heritability of 10.2%. 39 GWS loci associated with multisite CP. Genetic correlation with psychiatric, autoimmune, and anthropometric traits. Causal effect of multisite CP on MDD
Johnston et al.^64^	Multisite CP	GWAS	5 and 10 GWS loci associated with multisite chronic pain in men and women, respectively. Sex-specific gene associations and expression in dorsal root ganglion. Sex-specific association of multisite CP with MDD. Genetic correlation with a range of psychiatric and mood phenotypes
Kasher et al.^65^	CBP	CS/MR	RA, OP, CRP, BMI, age, and gender associated with CBP. Genetic correlation between CBP and RA and CRP and BMI. CRP causally predicts CBP. Pleiotropy seems to explain relationship between CBP and RA/OP
Khoury et al.^66^	Single and multisite CP	GWAS	23 GWS loci associated with multisite CP (none with single site CP) and 9 replicated in HUNT cohort. Axonogenesis in brain tissues is a major contributing pathway
Larvin et al.^70^	Painful gums	Longitudinal	Higher incidence of CVD and depression in painful gums compared with healthy controls. Increased risk of baseline → CVD → censor and baseline → metabolic → censor disease trajectory in painful gums. The former trajectory has increased the risk of mortality
Lobo et al.^73^	Chronic multisite musculoskeletal pain	CS/genetic association	Interaction between *FKBP5* rs3800373 risk variant and right hippocampal volume associated with chronic multisite musculoskeletal pain. This is mediated by severity of childhood trauma
Macfarlane et al.^75^	‘Any pain’, CP, and musculoskeletal pain	CS	Estimates of ‘any pain’, CP, and site-specific musculoskeletal pain prevalence similar between UK Biobank and MUSICIAN/NCDS cohorts
Macfarlane et al.^76^	CWP	Longitudinal/meta-analysis	CWP associated with excess all-cause mortality as well as excess cancer, cardiovascular, and respiratory-related deaths
Macfarlane et al.^77^	Opioid use	CS/longitudinal	5.5% of UK Biobank regularly using opioids. Opioid use is most common in groups of low socioeconomic status. Weak and strong opioids were associated with excess mortality
Macfarlane et al.^74^	(Chronic) Facial pain	CS	Overall prevalence of facial pain was 1.9%, of which 48% was chronic. Facial pain was more common in women, smokers and associated with psychological distress, low socioeconomic status, low alcohol consumption, and all types of regional pain
McIntosh et al.^81^	CP	CS/genetic association	PRS for MDD associated with CP in UK Biobank
McQueenie et al.^83^	CP/CWP	CS	Chronic pain is extremely common across a wide range of LTCs including migraine/headache, IBS, mental health conditions, and diseases of the digestive system. People with ≥4 LTCs 3 and 20 times more likely to have CP and CWP, respectively
Meng et al.^84^	Knee pain	GWAS	2 GWS loci (*GDF5* and *COL27A1*) associated with knee pain
Meng et al.^85^	Headache, facial, neck/shoulder, back, stomach/abdominal, hip and knee pain, and pain all over the body	Genetic association	Positive genetic correlation between all pain phenotypes and depressive symptoms, MDD, and neuroticism, except hip and knee pain
Meng et al.^86^	Neck and shoulder pain	GWAS	3 GWS loci associated with neck or shoulder pain. 2 loci (*FOXP2* and *LINC01572*) weakly replicated in an independent cohort. Genetic correlation between neck or shoulder pain and depression, insomnia, and neuroticism
Muralidharan et al.^88^	Multisite CP	Longitudinal/genetic association	Significant negative correlation between the number of chronic pain sites and age at death in men, but not women. *TP53* significantly associated with the number of chronic pain sites in women but not men
Nicholl et al.^89^	Multisite CP	CS	Individuals who report CP and multisite CP are more likely to have MDD and BD. Relationship between extent of CP and risk of MDD and BD
Nicholl et al.^90^	CP	CS	CP is more common, and depression is less common in Black and Asian ethnic groups compared with White. Association between presence and extent of CP and depression strongest in minority ethnic groups
Pan et al.^91^	Multisite musculoskeletal pain (hip, knee, back, and neck/shoulder pain)	CS	Greater number of painful sites consistently associated with poorer physical working capacity and low intensity physical activity compared with moderate or vigorous physical activity
Parisien et al.^92^	Acute back pain	CS	Elevated risk of acute back pain persistence in subjects taking NSAIDs
Patasova et al.^94^	Multisite CP and pain control medications (paracetamol, opioids, NSAIDs, and gabapentinoids)	CS/MR	Codeine, tramadol, paracetamol, ibuprofen, gabapentin, and pregabalin all individually associated with hyperopia. Causal effect of multisite CP on hyperopia
Rahman et al.^96^	CWP	GWAS	3 GWS loci (*RNF123*, *ATP2C1,* and *COMT*) associated with CWP. Partial genetic correlation between CWP and depressive symptoms, BMI, age of first birth, and years of schooling
Rönnegård et al.^99^	Acute pain, chronic localised pain, and CWP	Longitudinal	Increasing risk of composite CVD (myocardial infarction, stroke, heart failure, and cardiovascular mortality) in people with increasing pain duration and widespreadness
Rosoff et al.^100^	Prescription opioid use	MR	Evidence for potential causal associations between prescription opioid use and risk for MDD and ASRD. Genetic liability for MDD associated with increased risk of prescription opioid use
Shu et al.^103^	LBP	MR	Genetic correlation between LBP and insomnia and daytime sleepiness. Insomnia significantly associated with increased risk of LBP. No reverse causation nor a causal effect of daytime sleepiness on LBP
Slade et al.^106^	Facial pain	CS	Replication of considerable overlap of facial pain with pain in other parts of body. Greater association with headache and neck pain than pain below the neck
Suri et al.^114^	CBP	GWAS meta-analysis	GWS locus at *SOX5* associated and replicated with CBP. Two GWS loci (*CCDC26*/*GSDMC* and *DCC*) associated with CBP in meta-analysis
Tagliaferri et al.^115^	Acute back pain, chronic localised back pain, and CBP with other pain sites	CS	People with acute, chronic localised, and CBP with other pain sites have significant differences on brain structure and psychosocial and physical health states than people without pain
Tamosauskaite et al.^116^	CP	Genetic association	Homozygote *HFE* C282Y mutations (which is associated with excessive iron absorption) associated with CP in older men. *HFE* C282Y associated with knee, hip, and back pain in older women
Tang et al.^117^	BP	MR	Evidence for causal associations between serum iron, ferritin, and transferrin saturation and risk of back pain
Verma et al.^125^	Multisite CP	Genetic association	*EREG* (epiregulin) H2 haplotype protective for the presence of at least one CP site, H3 haplotype protective for chronic hip pain, and number of chronic pain sites
Walker-Bone et al.^129^	CWP	CS	A history of bone fracture is associated with increased risk of CWP in men and women
Zhang et al.^132^	Stomach/abdominal, multisite CP and neck/shoulder pain	GWAS	In patients with depression, *TRIOBP* associated with stomach/abdominal pain, *SLC9A9* associated with multisite CP, and *ADGRF1* associated with neck/shoulder pain
Zorina-Lichtenwalter et al.^133^	Multisite CP	Genetic association	*MC1R* variants involved in red hair associated with reduced count of pain conditions

(C)BP, (chronic) back pain; ABCB1, ATP binding cassette subfamily B member 1; ADGRF1, adhesion G protein-coupled receptor F1; AP, acute pain; ASRD, anxiety and stress-related disorders; ATP2C1, ATPase secretory pathway Ca2+ transporting 1; BD, bipolar disorder; BMI, body mass index; CCDC26, coiled-coil domain-containing 26; COL27A1, collagen Type XXVII alpha 1 chain; COMT, catechol-O-methyltransferase; COVID-19, coronavirus disease 2019; CP, chronic pain; CRP, C-reactive protein; CS, cross-sectional; CVD, cardiovasular disease; CWP, chronic widespread pain; DCC, deleted in colorectal cancer; EREG, epiregulin; FKBP5, FK506 binding protein 5; FOXP2, forkhead box protein P2; GDF5, growth differentiation factor 5; GSDMC, gasdermin C; GWAS, Genome-Wide Association Study; GWS, genome-wide significant; HFE, homeostatic iron regulator; HUNT, The Trøndelag Health Study; IBS, irritable bowel syndrome; LBP, low back pain; LINC01572, long intergenic nonprotein coding RNA 1572; LTC, long-term conditions; MC1R, melanocortin 1 receptor; MDD, major depressive disorder; MR, mendelian randomisation; MUSICIAN, managing unexplained musculoskeletal conditions using traditional and accessible new approaches; NCDS, National Child Development Study; NP, neuropathic pain; NSAID, nonsteroidal anti-inflammatory drug; OP, osteoporosis; PhWAS, Phenome-Wide Association Study; PRS, polygenic risk score; RA, rheumatoid arthritis; RNF123, ring finger protein 123; SLC9A9, solute carrier family 9 member A9; SNP, single nucleotide polymorphism; SOX5, SRY-box transcription factor 5; T2D, type 2 diabetes; TP53, tumor protein P53; TRIOBP, TRIO and F-actin binding protein.

Most pain studies conducted in UKB were cross-sectional because of the data being collected at a single time point. These have identified a wide range of associations with pain phenotypes including ethnicity,^90^ alcohol consumption,^8,74^ smoking,^74^ physical activity,^91^ low socioeconomic status,^1,74^ cardiovascular disease,^5^ type 2 diabetes (T2D),^5^ number of long-term comorbidities,^83^ adverse childhood experiences,^55^ depression,^89,90^ and bipolar disorder.^89^ They have also revealed that certain anatomical features are influential in pain. These include cam morphology (deformity of the femoral head–neck junction^37^), osteophytes^38,39^ and joint space narrowing^38^ with hip pain, bone fracture with chronic widespread pain (CWP),^96,129^ and differences in brain structure between acute back pain, chronic back pain, and chronic back pain occurring with other pain sites.^115^ UK Biobank has revealed that pain at specific sites, particularly facial pain, often overlaps with pain at other sites.^106^

The availability of self-reported medication (before UKB being linked to primary care records) has enabled the study of pain pharmacoepidemiology. Approximately 5.5% of people in UKB reported regular use of opioids (1.4% strong opioids and 4.2% weak opioids), which was found to be associated with low socioeconomic status and excess mortality.^77^ Strong opioid users (9.1%) were also more likely to die during follow-up than weak opioid users (6.9%) or nonusers (3.3%). The use of both opiate and NeuP pain medications with cardiometabolic medications was associated with obesity, increased waist circumference, and hypertension compared with taking cardiometabolic medications alone and the use of the antidiabetic drug metformin seemed to be protective of musculoskeletal pain.^27^ Common opioid (codeine and tramadol), nonsteroidal anti-inflammatory drug (NSAID) (ibuprofen), and NeuP medications were associated with far sightedness^94^ and people taking NSAIDs to treat back pain were more likely to report pain persistence than were those not treated with NSAIDs.^92^

Studies have also explored pain as a potential exposure for other clinical traits, particularly in longitudinal studies where the outcome has been measured at multiple time points. Using this approach, it has been demonstrated that CWP was associated with greater incidence of COVID-19 admission and mortality,^56^ as well as mortality relating to all-causes, cancer, respiratory disease, and cardiovascular disease.^76^ Chronic widespread pain and CP were also associated with cardiovascular disorders such as myocardial infarction, heart failure, and stroke,^99^ whereas a higher number of pain sites was associated with death at a younger age in men^88^ and all-cause mortality in both genders.^29^ The extensive data available in UKB allow researchers to adjust for a wide variety of potentially confounding factors, and this has been performed to a greater or lesser extent in the studies cited.

The availability of genome-wide genotyping data has advanced our understanding of genetic risk factors for pain and provided insights into the biological pathways involved. Novel genome-wide significant (GWS) genetic loci have been identified for back,^16,45,46,114^ knee,^84^ neck/shoulder,^86,132^ frozen shoulder,^53^ and stomach or abdominal pain^132^ as well as pain relating to oral inflammatory diseases,^62^ multisite CP,^63,64,66,132^ and CWP.^96^ These findings suggest a key role for genes involved in the central nervous system,^16,45,63,66^ dorsal root ganglion,^64^ and immune regulation.^62^ It has also highlighted some key differences in the genetics underpinning certain subsets of pain. For example, chronic back pain seems to be much more heritable than acute back pain (4.6% vs 0.8%).^16^ The same study identified 13 GWS loci associated with chronic back pain but none for acute back pain. Similarly, another study identified 23 GWS loci associated with multisite CP but none for single site CP.^66^ Sex-specific genetic risk factors have been identified in chronic back and multisite pain.^46,64^ Meanwhile, a separate study on chronic back pain identified 2 GWS loci in men but 7 in women.^46^

In addition to the identification of genetic risk factors, UKB genome-wide association study (GWAS) data have also been used to identify genetic correlation between different phenotypes. This is achieved by constructing polygenic risk scores (PRS) to summarise each participant's genetic predisposition for a given pain phenotype or by conducting linkage disequilibrium score regression (LDSR). These techniques have revealed, perhaps unsurprisingly, that there is strong genetic correlation between pain at different sites.^40^ Pain phenotypes also seem to have a shared genetic signature with a wide range of psychiatric and mood disorders such as depression, neuroticism, and sleep disorders,^45,86^ whereas shared genetic architecture seems to underpin the relationship between opioid cessation and CP, being a former drinker or being a former smoker.^32^

Finally, UKB GWAS data have been used to establish causal inference of nongenetic factors on pain phenotypes through Mendelian Randomisation. This technique uses known variation in a genetic marker and its influence on a particular trait (usually through GWAS) to interrogate the causal effect of an exposure on a particular outcome. As the genetic variants inherited by an individual are randomly assigned at conception and not subject to modification, it allows genetic markers to be used as a “proxy” for the exposure of interest, thus eliminating the risk of confounding or reverse causation. These studies have been able to establish a causal effect of C-reactive protein,^65^ insomnia^103^ and iron blood serum status^117^ with back pain, and diabetes with frozen shoulder.^53^ Furthermore, a bidirectional relationship was found to exist between insomnia and CP^21^ and between prescription opioid use and depression and anxiety disorders,^100^ whereas multisite CP was found to be a causative for major depressive disorder^63^ and far sightedness.^94^ By contrast, Mendelian Randomisation found no evidence for a causal effect of alcohol with CWP,^8^ obesity with frozen shoulder,^53^ or daytime sleepiness with lower back pain.^103^

## 3. DOLORisk

The approach to rephenotyping UKB participants for CP and NeuP was based on the experience of the DOLORisk consortium with phenotyping methods, in particular for population cohorts^93^ (Table 1).

**Figure 1.**
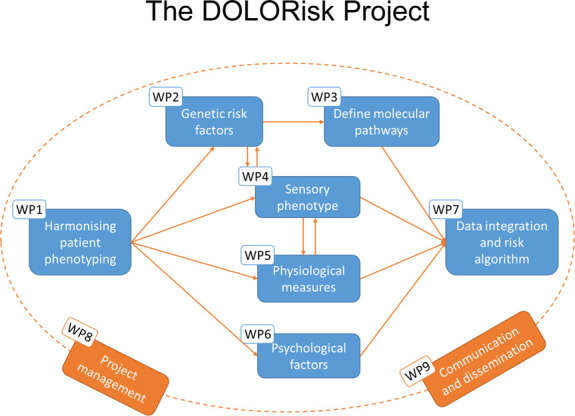
Illustration of the relationship between the different work packages in DOLORisk.

DOLORisk, funded by EU Horizon 2020, was the starting point of an effort to expand and improve the development of NeuP cohorts across Europe (including 11 participating centres). The aim was to develop clinical cohorts of sufficient scale to study the multiple risk factors and determinants for NeuP (genetic, clinical, and psychosocial), to understand how such factors interact, and to identify those individuals most at risk of NeuP (Fig. 1). Observational in design, it consisted of cross-sectional cohorts and longitudinal cohorts and included both participants from the community in whom outcome measures were captured using questionnaires and more specialised cohorts from secondary care who had detailed phenotyping.^93^ Participant recruitment occurred between 2015 and 2019.

The main longitudinal branch of the study consisted of 2 existing population cohorts: Generation Scotland (a family-based study^107^) and GoDARTS (Genetics of Diabetes Audit and Research in Tayside Study—focused on diabetes^57^), whose participants were contacted by the University of Dundee to be rephenotyped for NeuP (approximately 9,000 respondents at baseline^58^). Aarhus University and INSERM recruited smaller cohorts of participants scheduled to undergo chemotherapy, thoracic surgery, or breast cancer surgery. These also had a longitudinal design, assessing patients before and after surgery or chemotherapy. The other branch of DOLORisk was cross-sectional and consisted of cohorts of participants with a neuropathy assessed (with clinical history, examination, and specialised tests such as quantitative sensory testing [QST]) in research centres. Aetiologies of neuropathy included diabetic neuropathy, other polyneuropathies, postsurgical neuropathy, small fibre neuropathy, chemotherapy-induced neuropathy, rare pain disorders, and traumatic nerve injury. The total number of participants included in this deeply phenotyped cohort was in the region of 1500. An effort was made to include participants with the relevant predisposition such as diabetic neuropathy but without pain as a control group.

One of the ambitions of DOLORisk was to set standards for data collection and deep phenotyping of NeuP. All centres followed a common protocol as a means of harmonisation. This was developed around a core set of self-report questionnaires, to be administered to the population cohorts by mail, and based on recent international consensus on NeuP phenotyping which had been developed through systematic review, Delphi survey, and expert consensus meetings.^123^ Detailed aspects of the protocol including further questionnaires, clinical measures, and specialised tests were developed by consensus and finalised at a dedicated consensus meeting between all participating centres. Proposals were made with members of the consortium leading on their area of expertise, reviewing the literature, and unpublished data (for instance exploring the performance of a shorter version of the QST protocol) and then working to achieve a group consensus. In addition to the core questionnaires, the deeply phenotyped cohorts had an extended set of questionnaires, neurological examination, and physiological tests. The questionnaires captured information on demographics; medication; the presence, characterisation, and intensity of pain; pain interference; psychological and lifestyle factors; and quality of life. The choice of questionnaires was based on validation in CP/NeuP and the availability of relevant translations (details of the questionnaires and specialised tests can be found in Tables 1 and 2 of Ref. 93). Every participant had (or had previously provided) blood samples taken to perform genetic analyses. A subset of participants also provided a serum sample and a skin biopsy sample. Additional investigations included QST using a slightly shortened version of the German NeuP Consortium protocol,^98^ electrophysiology (including nerve conduction studies and nerve excitability testing^119^), conditioned pain modulation (CPM), and electroencephalography (EEG). The diagnosis of NeuP was graded based on the NeuPSIG algorithm,^44^ which sorts participants into 4 groups (unlikely, possible, probable, and definite NeuP) and in those participants with neuropathy diagnostic criteria were based on the Tesfaye criteria.^118^ The use of the same core questionnaires was to enable comparison between large population cohorts which have the advantage of scale but lack of in-depth phenotyping and the smaller cohorts recruited in secondary care. For clinical examination and the specialised tests, standard protocols were used including training (both in person and using videos) as well as regular review and feedback of data quality.

### 3.1. Caveats/limitations

(1) Generation Scotland has an overrepresentation of females, affluence, older age, and lower BMI and underrepresentation of comorbidities compared with the Scottish population. For example, 32% reported CP (2.7% severe) compared with 46% (5.7%) nationally.
(2) GoDARTS has an underrepresentation of non–Anglo-American ethnicities (0.3% vs 9.2%) and people who have never smoked (41.5% vs 48.1%) in the diabetic part of the cohort, compared with the Scottish T2D population in 2020 (https://www.diabetesinscotland.org.uk/wp-content/uploads/2022/01/Diabetes-Scottish-Diabetes-Survey-2020.pdf).
(3) Self-reported ethnicity was not recorded in DOLORisk limiting exploration of the impact of ethnicity on CP (this was due to national laws in one participating country preventing the recording of ethnicity data).
(4) Most cohorts which underwent deep phenotyping in DOLORisk were cross-sectional rather than longitudinal limiting our ability to establish causality in the relationship between risk factor(s) and CP. This will be partly addressed by PAINSTORM (see below) which will collect follow-up data on these cohorts within the United Kingdom.

### 3.2. Outputs

DOLORisk ran from 2015 to 2020, and consequently studies are now beginning to be published (Table 3). The first study to emerge was a GWAS meta-analysis of Generation Scotland and GoDARTS (using a questionnaire-based phenotype identifying NeuP of any aetiology) together with UKB (using a phenotype based on self-reported medication^124^). This revealed a novel genome-wide significant locus at the mitochondrial phosphate carrier gene *SLC25A3* and a suggestive locus at the calcium-binding gene *CAB39L*. In parallel with this study, the questionnaire-based data in Generation Scotland were used to construct longitudinal environmental risk models for onset and resolution of NeuP, which were then validated in GoDARTS.^59^ These models demonstrated the importance of psychological, social, lifestyle, and personality factors in predicting NeuP outcomes. The GoDARTS cohort was also used as a validation cohort in a study which trained machine learning models that can classify people with diabetic peripheral neuropathy into those with and without pain.^6^ These models were developed in deeply phenotyped cohorts recruited from the University of Oxford, Technion-Israel Institute of Technology, and Imperial College London and again highlighted the importance of personality, psychological, and quality of life factors in predicting pain. Other important predictors identified were levels of glycosylated haemoglobin (HbA1c), age, and BMI. It is hoped that eventually, these models can be used in a clinical setting to help improve prevention, diagnosis, and treatment for patients.

**Table 3:** Publications on neuropathic pain from the DOLORisk study.

Study	Pain phenotype	Design	Main finding
Baskozos et al.^6^	Diabetic peripheral neuropathy	CS	Machine learning techniques demonstrated that HRQoL, personality traits, HbA1c, depression, anxiety, age, and BMI were the most powerful predictors of the presence of in pain people with diabetic peripheral neuropathy
Bennedsgaard et al.^11^	Chemotherapy-induced peripheral neuropathy in patients with breast cancer referred for surgery	Longitudinal	Polyneuropathy and pain symptoms more common in patients treated with chemotherapy than those without. In people treated with chemotherapy, pain in the feet was less common than pain at the surgical site but was more intense
Granovsky et al.^52^	Diabetic polyneuropathy	CS	Heat pain CPM was more efficient in people with painful vs painless diabetic polyneuropathy. Efficient heat pain CPM associated with greater pain intensity in previous 24 h and greater loss of mechanical sensation.
Hébert et al.^59^	General NP	Longitudinal	Multivariable risk models demonstrate that psychosocial and lifestyle factors predict the onset and resolution of neuropathic pain. The models demonstrated adequate discrimination and clinical utility over a range of risk thresholds
Themistocleous et al.^119^	Diabetic/chemotherapy-induced distal symmetrical polyneuropathy	CS	There were no significant differences in sensory and motor axonal excitability measures between patients with painful and painless diabetic/chemotherapy-induced peripheral neuropathy
Topaz et al.^120^	Diabetic polyneuropathy	CS	Advanced predictive analysis demonstrated that resting-state electroencephalography–based functional brain activity (cortical functional connectivity) can discriminate between painful and painless diabetic polyneuropathy
Veluchamy et al.^124^	General NP	GWAS meta-analysis	1 GWS significant locus (*SLC25A3*) and one suggestive locus (*CAB39L*) associated with NP. Gene expression in NP-associated brain and DRG tissue. Previously reported genetic variants failed to replicate

BMI, body mass index; CAB39L, calcium binding protein 39 like; CPM, conditioned pain modulation; CS, cross-sectional; DRG, dorsal root ganglion; GWAS, Genome-Wide Association Study; GWS, genome-wide significant; HbA1c, glycated haemoglobin; HRQoL, health-related quality of life; SLC25A3, NP, neuropathic pain; solute carrier family 25 member 3.

Further studies have been conducted on patients with diabetic polyneuropathy in more deeply phenotyped cohorts. For example, in a cohort recruited by Technion-Israel, both traditional and machine learning predictive techniques were used to analyse brain activity through EEG data.^120^ This analysis revealed that people with painful diabetic polyneuropathy had significantly greater resting-state cortical functional connectivity than people with painless diabetic polyneuropathy and that EEG-based brain activity could be a powerful biomarker than can accurately discriminate between the 2 groups. Another study using the cohort from Technion and a cohort recruited by Imperial College London found that people with painful diabetic polyneuropathy had more efficient CPM to heat stimuli applied to the forearm than those with painless diabetic polyneuropathy.^52^ This was the first comparison of CPM in painful vs painless diabetic neuropathy. Previous studies had compared groups of patients with CP to healthy controls and so would not take into account neuropathy induced damage to sensory afferents. Conditioned pain modulation heat stimuli efficiency was also correlated with greater pain intensity in the previous 24 hours and greater loss of mechanical sensation. One possible explanation for the more efficient CPM to heat stimuli in those with painful diabetic polyneuropathy may relate to neuropathy at the site of stimulation used in the protocol (and not only as a consequence of descending pain modulation). In light of this, new protocols in which stimuli are given to sites unaffected by neuropathy are needed. Finally, a study exploring the use of nerve excitability (using threshold tracking) as a biomarker in patients with diabetic and chemotherapy-induced peripheral neuropathy found that there was no difference in axonal excitability relating to large, myelinated fibers between those with pain and those without pain.^119^ However, because nociceptors are generally unmyelinated and, therefore, not assessed using this technique, these findings suggest that alternative techniques such as microneurography (which specifically examines small fibres) should be used to explore the relationship between neuron excitability and NeuP.

Separately, a longitudinal investigation of patients with breast cancer referred for surgery found that, in the group who had received chemotherapy, pain at the surgical site was more prevalent than pain in both feet (59% vs 30%).^11^ However, the pain in the feet was rated as more intense and with more daily life interference than pain in the surgical area. Furthermore, the prevalence of pain in both feet was greater in those who had pain in the surgical area, compared with those who did not have pain in the surgical area (40% vs 17%).

Analysis of the DOLORisk cohort is ongoing especially in relation to the deeply phenotyped cohorts including sensory profiles (determined using QST) and genomics.

## 4. PAINSTORM

Following on from DOLORisk, the PAINSTORM project (Partnership for Assessment and Investigation of Neuropathic Pain: Studies Tracking Outcomes, Risks and Mechanisms) was funded by the UK's Advanced Pain Discovery Platform (APDP),^4^ beginning in 2021. PAINSTORM will follow-up the DOLORisk diabetic population cohort in Dundee (GoDARTS), the diabetic and idiopathic neuropathy cohorts at Oxford and Imperial, and further expand the Oxford rare phenotypes cohort. It will also include new cohorts of people receiving chemotherapy, people with HIV and HTLV-1, and will use the newly available CP data in UKB. The aim of PAINSTORM is to collect more longitudinal data, especially in the deeply phenotyped cohorts, to define the risk factors and pathophysiological drivers of NeuP. Patient partners contributed directly to the consensus meeting and to the development of the PAINSTORM protocol and both of these are very similar to those in DOLORisk (Table 1). Based on feedback from patient partners and past experience, a few questionnaires were added (ethnicity, PROMIS Emotional Support, PROMIS Instrumental Support, bespoke items related to pain management, and the lived experience of having NeuP), substituted (PROMIS Pain Interference was replaced with PROMIS Ability to Participate in Social Roles and Activities; the IPIP items for Emotional Stability were replaced with the 7-item State Optimism Measure), or removed (PainDETECT). The inclusion of patient partners in PAINSTORM (form the application stage) has shaped our understanding of the issues that matter to people living with NeuP, the lived experience of NeuP, and the acceptability of measures to assess NeuP. The specialised investigation techniques in PAINSTORM differ slightly to DOLORisk: Nerve excitability testing (which we found did not discriminate painful from painless neuropathy in the DOLORisk study^119^) makes way for microneurography^102,122^; CPM was omitted as our data from the DOLORisk project suggest that an improved CPM protocol, to be applied and validated in the context of neuropathy, is required^52^; EEG was not included because as yet the technology for undertaking this at scale is not available; and some participants will take part in imaging studies of the brain, the spinal cord, and the peripheral nervous system. Genetic analysis is likely to include technology advances in both sequencing and analysis for much more comprehensive genomic assessment such as whole genome sequencing.

## 5. Caveats and lessons learned

Other important and relevant pain cohorts exist (such as OPPERA examining painful temporomandibular disorder^43,104,105^), and we have only described 3 in order that they can be discussed in detail. UK Biobank, DOLORisk, and PAINSTORM cohorts include a specific focus on (neuropathic) pain phenotypes. Some other cohorts include a few pain questions, but pain is not the focus, and these questions are almost incidental, although they can have value. For example, the English Longitudinal Study of Ageing (ELSA) included a single question about pain (“Are you often troubled by pain?”—yes/no), which allowed relatively detailed analysis of associations between pain and mortality.^109^ In one sense, this caveat could even be true of UKB at baseline, which used an untried, unvalidated, nonstandard set of relatively superficial questions. This has allowed a good number of studies to be published (Table 2), and their success may rely on sample size and consequent power, rather than on the precision or validity of the definitions. Not until the rephenotyping exercise, described above (UKB CP) were there validated, standard pain, and relevant associated questionnaires included.

The 3 cohorts we have described deliberately used similar, harmonised approaches to phenotyping pain. Generally, when looking at different research cohorts, we find that a lack of agreed approaches to phenotyping CP means that we cannot compare outputs from different cohorts. For example, in a systematic review of studies examining genetic factors associated with NeuP, we found 29 studies, identifying 28 genes, but none used the same approach to phenotyping, and few single genes were identified by more than one study. This means that we cannot understand whether differences between studies are the result of actual differences between study populations or artefacts of differential phenotyping. One study, for example, found that associations between CP and mortality depended on how the pain was phenotyped.^108^ This lack of harmonisation also prevents meta-analysis.

To surmount these phenotyping/case definition differences, we need the following:

(1) A series of studies exploring the effects that differences in case definition have in identifying subsamples with and without CP; eg, demographic and clinical differences/similarities between “cases” in different cohorts. Macfarlane et al.^75^ did explore this in relation to the original UKB pain phenotype, comparing prevalence and associations with those identified in other pain cohorts, and we have performed similar in relation to UKB CP (paper in press). Although the results were reassuring, more such analysis is called for.
(2) Harmonised case definitions/phenotyping moving forward, such as that agreed for genetic studies of NeuP (NeuroPPIC^123^). In addition to allowing comparison and meta-analysis (retrospective studies), this approach will also allow prospectively assembled collaborative cohorts, with enhanced power. This has been our philosophy with UKB CP, DOLORisk, and PAINSTORM.

A major issue with the cohorts we have described, and with every other cohort, is their representativeness. Even a very large cohort such as UKB can only reflect the population from which it was drawn. As noted above, in UKB's case, this population comprised adults aged 40 to 69 at recruitment,^112^ and underrepresents people from more socioeconomically deprived areas, as well as people who are obese, smoke, drink alcohol, self-report certain health conditions, and ethnic minorities.^48^ Although this allows assessment of relationships between exposures and outcomes, it limits findings relating to incidence and prevalence and to exposures/outcomes that are rare in the cohort. Similar constraints apply to the studies contributing to DOLORisk (including GS^107^ and GoDARTS^57^) and will also apply to PAINSTORM. A key is to focus on the strengths and unique selling points of the cohort; eg, a family study allows efficient measurement of heritability. It is also important to measure, understand, and, if possible, account or adjust for relevant differences between the cohort and target populations. Strategies also need to be developed to improve representation in groups that are traditionally underrepresented in research. Potential approaches for improving representativeness include expanding recruitment strategies, to include for example face-to-face, email, and postal invitations, through primary and specialist care. In addition, dedicating research resources into advertising through local/national business organisations, radio, newspapers, and social media can increase uptake and help to oversample these “hard-to-reach” groups. The involvement of people living with pain, for instance coworking with charities and community organisations can help with dissemination strategies and generate general awareness of studies. An example of an initiative that has successfully used these approaches in the Scottish Health Research Register (SHARE).^82^ However, there are certain situations in which a representative sample may not be necessary, for example, if an investigator wants to study a particular population subgroup.^97^

For practical reasons, large population studies generally only allow brief questionnaire measures, often purporting to represent complex multidimensional phenomena (such as pain) in summary numerical terms, often with continuous scales or categorical coding. Although these questionnaires are (1) usually validated in their development stages by comparison with more sophisticated and detailed assessments and (2) often supplemented in related studies by more detailed measurement/interviews with subsamples, they cannot tell the full story of what is being measured. This issue has long been recognised (eg, by Macnaughton^79^), and recent discussions with our patient partners on PAINSTORM confirm the issue, and the frustration it causes to people completing the questionnaires. Although we should continue to measure as accurately as possible, using questionnaires with maximum validity and reliability, we should also work with people living with pain to develop more realistic and satisfactory ways of assessing complex health and psychosocial issues at scale.

## 6. Future prospects

Alternative approaches to cohort recruitment and assessment include the use of routine clinical data, without the direct involvement of individuals (although with appropriate ethical and governance approvals in place). This approach has also been recommended for clinical trials in CP.^101^ For example, in the United Kingdom, GP-held primary care records offer the opportunity to identify relevant individuals through clinical diagnostic codes or prescribing. These have been used to recruit study participants with CP^22,80^ but require detailed validation and assessment of sensitivity, specificity, and positive/negative predictive values. The authors (BHS and DW) are currently developing this in 2 funded studies at the population level. Routine clinical data can also augment research-derived data arising from new and existing cohorts, through data linkage, noting the need for data security and confidentiality. As noted, UKB data are now linked to primary healthcare records for most participants. Advantages of linkage to routine data include comprehensiveness, representativeness, and low cost. Disadvantages include relatively poor-quality data and the complexity of data available, as well as the need to secure different approvals before data can be accessed and linked.

Development, harmonisation, meta-analysis, and data linkage of CP cohorts require, among other factors, high-quality data storage, management, and access. Also funded through the APDP, Alleviate is the Research Data Hub that will provide a platform for pain data for researchers around the world.^2^ Initially focusing on APDP consortia (including PAINSTORM) and projects, Alleviate will also store or allow access to other relevant datasets (including UKB CP and DOLORisk). These data sets will be findable, accessible, interoperable, and reusable (FAIR), and the Hub will be comparable with those already in existence for respiratory datasets (BREATHE^19^) and COVID-19 (CO-CONNECT^31^). Investment in data hubs such as Alleviate from researchers and funding bodies is key to expanding these cohorts on a global scale and integrating efforts around data harmonisation. Such investment can be encouraged and promoted through organisations such the International Association for the Study of Pain.

Meanwhile, we will continue our research with the above cohorts, both through further analysis of DOLORisk and UKB CP, and through development of PAINSTORM. Importantly, this will include collaboration with colleagues and cohorts elsewhere, including other APDP consortia and projects,^4^ with whom PAINSTORM is harmonising as much as possible. The scheduled follow-up of UKB CP phenotyping promises exciting approaches to longitudinal research on CP at large scale.

## 7. Conclusions

Large national level biobanks such as UKB have already provided important insights into the pathophysiology of CP; these are likely to become more robust with the greater precision of recently augmented pain phenotyping and longitudinal outcomes. There are exciting prospects ahead with the greater integration of GP records, new genetic technologies (such as whole genome sequencing), and the brain imaging of 100,000 participants in UKB. Making sense of such complex, multimodal data sets will require advanced analytics, including machine learning approaches. Although the scale of UKB has undoubted advantages, pain remains a subjective phenomenon which is difficult to capture using a limited number of questionnaires. This is particularly important in conditions such as NeuP where clinical assessment (including examination) is required to reach a robust case definition. This means that there is still an important place for smaller deeply phenotyped cohorts in which the link between a predisposing aetiology, CP, and biomarkers can be studied in detail. Such cohorts can also be used (while working with patient partners) to find new ways to assess pain and the functional impact of pain which can then be iteratively fed back to national level cohorts. The pharmaceutical industry is increasingly orienting analgesic drug discovery to targets in which there is human data validating the molecular target. Our hope is that integration of “big pain data” in humans with the rapid advances in cellular transcriptomics and neural circuit level approaches in animal models will facilitate the desperately needed development of novel analgesics, among other important advances.

## Disclosures

The authors have no conflict of interest to declare.
